# Supplementary material for: Reactivatable stimulated emission depletion microscopy using fluorescence-recoverable nanographene
Source: Nat Commun. 2025 Feb 4;16:1341. doi: 10.1038/s41467-025-56401-z (PMC11794581; doi:10.1038/s41467-025-56401-z)
Supplement: Supplementary file 1 — Supplementary Information [file 41467_2025_56401_MOESM1_ESM.pdf]

Supplementary Information for

Reactivatable stimulated emission depletion microscopy using fluorescence-recoverable nanographene

Qiqi Yang, Antonio Virgilio Failla, Petri Turunen, Ana Mateos-Maroto, Meiyu Gai, Werner Zuschrattter, Sophia Westendorf, Márton Gelléri, Qiang Chen, Goudappagouda, Hao Zhao, Xingfu Zhu, Svenja Morsbach, Marcus Scheele, Wei Yan, Katharina Landfester, Ryota Kabe*, Mischa Bonn*, Akimitsu Narita*, Xiaomin Liu*

*Corresponding authors: ryota.kabe@oist.jp (R.K.); bonn@mpip-mainz.mpg.de (M.B.); akimitsu.narita@oist.jp (A.N.); Liuxiaomin@mpip-mainz.mpg.de (X.L.).

The PDF file includes:

Supplementary Note 1
Supplementary Figures 1 to 24

Other Supplementary Materials for this manuscript include the following:
Supplementary Movies 1 and 2

Contents

Supplementary Note 1: Detailed microscope settings for figures in the main text 3

Supplementary Figure 1: STED light does not photo-bleach DBOV-Mes 8

Supplementary Figure 2: Photo-bleaching properties of DBOV by excitation laser 9

Supplementary Figure 3: Representative line profiles from images in Fig. 1b-d 10

Supplementary Figure 4: Possible ionization mechanisms 11

Supplementary Figure 5: UV-vis-NIR absorption spectra of DBOV-Mes and DBOV-Mes$^{\bullet+}$ 12

Supplementary Figure 6: EMAS measurement of a DBOV-Mes film 13

Supplementary Figure 7: Calculated 2D STED intensity distributions 15

Supplementary Figure 8: Measured leftover fluorescence ratio dependent on the confocal excitation laser density over time 16

Supplementary Figure 9: Plot (ln–ln) of fluorescence ratio against power density 16

Supplementary Figure 10: Two-photon fluorescence under high STED beam peak intensity 17
Supplementary Figure 11: Fluorescence retention after different STED power densities scanning 17
Supplementary Figure 12: Fluorescence retention after long term deactivation and reactivation cycles 18
Supplementary Figure 13: Fluorescence decreases in long-term STED doughnut beam (only 775 nm) scanning 19
Supplementary Figure 14: STED imaging mode used to characterize the reactivation properties 20
Supplementary Figure 15: Reactivation measured in air, H_2O and PBS 21
Supplementary Figure 16: Combined illumination of the excitation and STED beams has negligible photo-bleaching effect on the sample 22
Supplementary Figure 17: Reactivation by NIR light at 730 nm 23
Supplementary Figure 18: Reactivation by shorter visible light at 405 nm and spontaneously decay 24
Supplementary Figure 19: Photostability from two 3D STED imaging 25
Supplementary Figure 20: Schematic diagram of liposome preparation 26
Supplementary Figure 21: Size and zeta potential characterization of DBOV-Mes-labeled freshly prepared liposomes 26
Supplementary Figure 22: Schematic diagram of liposome immobilization 27
Supplementary Figure 23: STED images of deformed liposomes 28
Supplementary Figure 24: Non-deconvolved STED images corresponding to Fig. 5 29
Supplementary references 29

Supplementary Note 1: Detailed microscope settings for figures in the main text

Figure 1 (Leica Stellaris 8 STED microscope):

b-**d**, Images acquired by the following 5 steps (of one same imaging position):

Step 1, 3 and 5, confocal images were acquired before deactivation, after deactivation and after reactivation, respectively. One confocal image using 561 nm (0.22 mW) as excitation, 512 pixel X 512 pixel, 0.076 µm per pixel, scan speed 100 Hz, pixel dwell time 15.3875 µs, line accumulation of 2, HyD X4 (600 nm – 748 nm). Images from step 1, 3 and 5 are the left, middle and right images in Fig. 1b respectively.

Step 2, confocal images (small, center of image of step 1) were acquired for deactivation. Confocal images (100 frames) were acquired using 561 nm (0.22 mW) as excitation, 64 pixel X 64 pixel, 0.109 µm per pixel, scan speed 100 Hz, pixel dwell time 102.375 µs, line accumulation of 2, total collection time is 137.041 s.

Step 4, images (the same imaging area of step 2) scanned by STED doughnut beam were acquired for reactivation. Images (5 frames) were acquired using only the 775 nm (600 mW) beam (doughnut shape), 64 pixel X 64 pixel, 55.916 nm per pixel, scan speed 100 Hz, pixel dwell time 102.375 µs, line accumulation of 2, total reactivation time is 5.614 s. Notes: The 100% laser power in this experiment was 600 mW.

Figure 2 (Leica Stellaris 8 STED microscope):

b, Images acquired by the following 3 steps (of one same imaging position):

Step 1 and 3, confocal images were acquired for measuring fluorescence intensity – before and after deactivation, respectively. One confocal image using 561 nm (0.22 mW) as excitation, 512 pixel X 512 pixel, 0.076 µm per pixel, scan speed 100 Hz, pixel dwell time 15.3875 µs, line accumulation of 2, HyD X4 (600 nm – 748 nm).

Step 2, confocal images (small, center of image of step 1) were acquired for deactivation. Confocal images (150 frames) were acquired using 561 nm (0.19 mW) as excitation, 64 pixel X 64 pixel, 0.109 µm per pixel, scan speed 100 Hz, pixel dwell time 102.375 µs, line accumulation of 2, HyD X4 (600 nm – 748 nm). Total collection time is 203.147 s.

The power dependence experiments were done by keeping the same setting, only varying excitation power (0.19, 0.15, 0.12, 0.07, 0.05 mW), and choosing a new imaging position at each excitation power. Under each excitation power, 3 or 4 times experiments were repeated at different imaging positions. Data analysis was done as in Methods and using equations in Methods. Error bars represent standard deviations calculated from 3 or 4 measurements.

c, Images acquired by the following 5 steps (of one same imaging position):

Step 1, 3 and 5, confocal images were acquired for measuring fluorescence intensity – before deactivation, after deactivation and after reactivation, respectively. One confocal image using 561 nm (0.22 mW) as excitation, 512 pixel X 512 pixel, 0.076 μm per pixel, scan speed 100 Hz, pixel dwell time 15.3875 μs, line accumulation of 2, HyD X4 (600 nm – 748 nm).

Step 2, confocal images (small, center of image of step 1) were acquired for deactivation. Confocal images (150 frames) were acquired using 561 nm (0.19 mW) as excitation, 64 pixel X 64 pixel, 0.109 μm per pixel, scan speed 100 Hz, pixel dwell time 102.375 μs, line accumulation of 2, HyD X4 (600 nm – 748 nm). Total collection time is 203.146 s.

Step 4, images (the same imaging area of step 2) scanned by STED doughnut beam were acquired for reactivation. Images (5 frames) were acquired using only 775 nm (270 mW) beam, 64 pixel X 64 pixel, 0.109 μm per pixel, scan speed 100 Hz, pixel dwell time 102.375 μs, line accumulation of 2, total collection time is 5.454 s.

The power dependence experiments were done by keeping the same setting only varying STED beam power (270, 220, 194, 167, 140, 112, 84, 56 and 27 mW) and choosing a new imaging position at each STED beam power. Under each STED power, 3 or 4 times experiments were repeated at different imaging positions. Notes: Due to system reasons, the STED power decreased slowly over months, and the 100% laser power in this experiment was 270 mW. Data analysis was done as in Methods and using equations in Methods. Error bars represent standard deviations calculated from 3 or 4 measurements.

d, For the cycles of short-term deactivation-reactivation, images were acquired by the following 5 steps (of one same imaging position):

Step 1, 3 and 5, confocal images were acquired for measuring fluorescence intensity – before deactivation, after deactivation and after reactivation, respectively. One confocal image using 561 nm (0.22 mW) as excitation, 512 pixel X 512 pixel, 0.076 μm per pixel, scan speed 100 Hz, pixel dwell time 15.3875 μs, line accumulation of 2, HyD X4 (600 nm – 748 nm).

Step 2, confocal images (small, center of image of step 1) were acquired for deactivation. One confocal image (1 frame) was acquired using 561 nm (0.14 mW) as excitation, 64 pixel X 64 pixel, 0.109 μm per pixel, scan speed 100 Hz, pixel dwell time 102.375 μs, line accumulation of 2.

Step 4, images (the same imaging area of step 2) scanned by STED doughnut beam were acquired for reactivation. One image (the same imaging area of step 2) was

acquired using only the 775 nm (140 mW) beam (doughnut shape), 64 pixel X 64 pixel, 55.916 nm per pixel, scan speed 100 Hz, pixel dwell time 102.375 μs, line accumulation of 2.

After measuring the first set (steps 1 to 5), loop of 6 cycles between steps 2 to 5 were acquired at the same imaging position. For the cycles without reactivation, images were acquired as above but without step 4.

e, For the cycles of long-term deactivation-reactivation, images were acquired by the following 5 steps (of one same imaging position):

Step 1, 3 and 5, confocal images were acquired for measuring fluorescence intensity – before deactivation, after deactivation and after reactivation. One confocal image using 561 nm (0.22 mW) as excitation, 512 pixel X 512 pixel, 0.076 μm per pixel, scan speed 100 Hz, pixel dwell time 15.3875 μs, line accumulation of 2, HyD X4 (600 nm – 748 nm).

Step 2, confocal images (small, center of image of step 1) were acquired for deactivation. Confocal images (100 frames) were acquired using 561 nm (0.22 mW) as excitation, 64 pixel X 64 pixel, 0.109 μm per pixel, scan speed 100 Hz, pixel dwell time 102.375 μs, line accumulation of 2, total deactivation time is 134.976 s.

Step 4, images (the same imaging area of step 2) scanned by STED doughnut beam were acquired for reactivation. Images (25 frames) were acquired using only the 775 nm (140 mW) beam (doughnut shape), 64 pixel X 64 pixel, 0.109 μm per pixel, scan speed 100 Hz, pixel dwell time 102.375 μs, line accumulation of 2, total reactivation time is 32.721 s.

After measuring the first set (steps 1 to 5), loop of 4 cycles between steps 2 to 5 were acquired. Error bars represent standard deviations calculated from 4 measurements.

Figure 3:

a,**b**, Images acquired by the following 4 steps:

Step 1 and 4, confocal images were acquired for measuring fluorescence intensity – before and after confocal and STED imaging, respectively. One confocal image using 561 nm (0.22 mW) as excitation, 512 pixel X 512 pixel, 0.076 μm per pixel, scan speed 100 Hz, pixel dwell time 15.3875 μs, line accumulation of 2, HyD X4 (600 nm – 748 nm).

Step 2, confocal images (small, left region of image of step 1) were acquired. Move the center 10 um to the left relative to Step 1. Confocal images (5 frames) were acquired using 561 nm (0.22 mW) as excitation, 64 pixel X 64 pixel, 0.109 μm per pixel, scan speed 100 Hz, pixel dwell time 102.375 μs, line accumulation of 2, HyD X4 (600 nm – 748 nm). Total imaging time is 5.454 s.

Step 3, STED images (the same imaging size of images of step 2, right region of image of step 1) were acquired. Move the center 10 um to the right relative to Step1. STED images (5 frames) were acquired using 561 nm (0.19 mW) excitation beam and 775 nm (270 mW) STED beam, 64 pixel X 64 pixel, 0.109 µm per pixel, scan speed 100 Hz, pixel dwell time 102.375 µs, line accumulation of 2, HyD X4 (600 nm – 748 nm). Total imaging time is 5.454 s.

Notes: Due to system reasons, the STED power decreased over months, and the 100% laser power in this experiment was 270 mW.

c, Comparison of fluorescence bleaching of confocal and STED imaging were done by keeping the same method in Fig. 2D only varying imaging frames/time. Total imaging time is 1.363 s (2 frames), 2.727 s (3 frames), 4.090 s (4 frames), 5.454 s (5 frames), 12.271 s (10 frames), 25.904 s (20 frames), 66.807 s (50 frames), 134.976 s (100 frames), and 407.657 s (300 frames). Under each excitation power, 3 or 4 times experiments were repeated at different imaging positions. Data analysis was done as in Methods. Error bars represent standard deviations calculated from 3 or 4 measurements.

d, Images acquired by the following 3 steps (of one same imaging position):

Step 1 and 3, confocal images were acquired for measuring fluorescence intensity – before and after STED imaging, respectively. One confocal image using 561 nm (0.22 mW) as excitation, 512 pixel X 512 pixel, 0.076 µm per pixel, scan speed 100 Hz, pixel dwell time 15.3875 µs, line accumulation of 2, HyD X4 (600 nm – 748 nm).

Step 2, STED images (small, center of image of step 1) (150 frames) were acquired using 561 nm (0.22 mW) excitation beam and 775 nm (335 mW) STED beam, 64 pixel X 64 pixel, 0.109 µm per pixel, scan speed 100 Hz, pixel dwell time 102.375 µs, line accumulation of 2, HyD X4 (600 nm – 748 nm). Total imaging time is 203.147 s.

The power dependence experiments were done by keeping the same setting only varying STED beam power (335, 270, 207, 138, 69 and 35 mW) and choosing a new imaging position at each STED power. Under each STED power, 3 or 4 times experiments were repeated at different imaging positions. Notes: Due to system reasons, the STED power decreased over months, and the 100% laser power in this experiment was 335 mW. Data analysis was done as in Methods and using equations in Methods. Error bars represent standard deviations calculated from 3 or 4 measurements.

e, Images acquired by the following 5 steps (of one same imaging position):

Step 1, 3 and 5, confocal images were acquired for measuring fluorescence intensity – before deactivation, after deactivation and after reactivation, respectively. One confocal

image using 561 nm (0.22 mW) as excitation, 512 pixel X 512 pixel, 0.076 µm per pixel, scan speed 100 Hz, pixel dwell time 15.3875 µs, line accumulation of 2, HyD X4 (600 nm – 748 nm). Images from step 1, 3 and 5 are similar to the left, middle and right images in Fig. 1b respectively.

Step 2, confocal images (small, center of image of step 1) were acquired for deactivation. Confocal images (100 frames) were acquired using 561 nm (0.22 mW) as excitation, 64 pixel X 64 pixel, 0.109 µm per pixel, scan speed 100 Hz, pixel dwell time 102.375 µs, line accumulation of 2, total collection time is 134.977 s.

Step 4, images (the same imaging area of step 2) scanned by STED doughnut beam were acquired for reactivation. Images (10 frames) were acquired using only 775 nm (450 mW) beam, 64 pixel X 64 pixel, 55.916 nm per pixel, scan speed 100 Hz, pixel dwell time 102.375 µs, line accumulation of 2, total collection time is 12.270 s.

Samples were prepared as described in Methods, 10µL of H_2O or PBS was dropped on the sample surface for imaging. Under each condition, 3 or 4 times experiments were repeated at different imaging positions. Notes: Due to system reasons, the STED power decreased over months, and the 100% laser power in this experiment was 450 mW. Data analysis was done as in Methods. Error bars represent standard deviations calculated from 3 or 4 measurements.

Figure 4:

a-**d**, Leica Stellaris 8 STED microscope: 561 nm (0.22 mW) as excitation, 775 nm (450 mW) STED beam, 512 pixel X 512 pixel, 0.015 µm per pixel, scan speed 400 Hz, pixel dwell time 3.1625 µs, line accumulation of 2, HyD X4 (586 nm – 748 nm).

f-**h**, Leica TCS SP8 STED microscope: 561 nm (AOTF setting: 40.52%) as excitation, 775 nm (AOTF setting: 86.03%) STED beam, 2048 pixel X 2048 pixel, 0.019 µm per pixel, 54 steps for z stack with 0.156 µm per step, scan speed 400 Hz, pixel dwell time 0.3 µs, line accumulation of 8, HyD 2 (600 nm – 748 nm).

Figure 5 (Abberior Expert Line microscope):

STED 2D images were acquired with x,y voxel to be 20 x 20 nm, STED 3D pictures were took with x,y,z voxel to be 40 x 40 x 40 nm, respectively.

Supplementary Figure 1: STED light does not photo-bleach DBOV-Mes

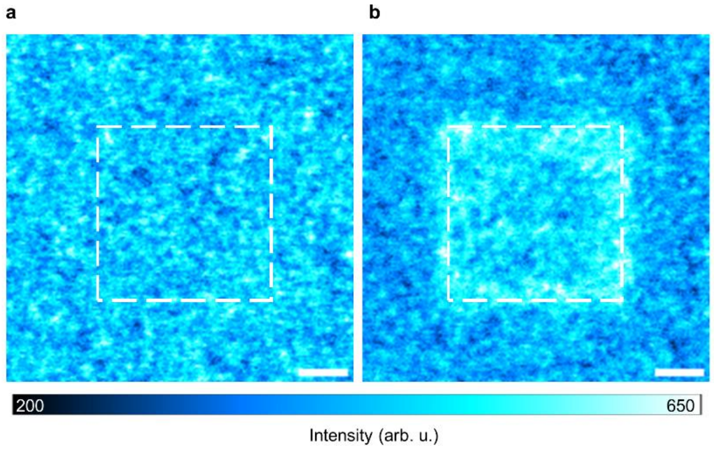

Images show STED light does not photo-bleach DBOV-Mes. Confocal images of DBOV-Mes on coverslip surface before (**a**) and after (**b**) scanned by STED doughnut beam for 100 frames. Measured with Leica Stellaris 8 STED microscope. White dashed square region: STED beam scanned area. STED laser: 775 nm, 140 mW. Scales, 2 µm. The color bar represents a linear scale. The fluorescence still kept 110% (average of 4 with standard deviations of 8%) after STED doughnut beam scanned for 100 frames. The fluorescence intensity after scanned by the STED beam was even higher than in the first image. This is due to factors such as ambient light which is difficult to avoid and the beam excitation that is required to find the focal plane and select the target imaging area before imaging. Therefore, before taking the first confocal image, some DBOV-Mes molecules may already be in the fluorescent OFF state.

These images were acquired by the following 3 steps (of one same imaging position):

Step 1 and 3, confocal images were acquired before and after scanned by STED doughnut beam. One confocal image using 561 nm (0.22 mW) as excitation, 512 pixel X 512 pixel, 0.076 µm per pixel, scan speed 100 Hz, pixel dwell time 15.3875 µs, line accumulation of 2, HyD X4 (600 nm – 748 nm).

Step 2, Scanned by only 775 nm STED doughnut beam (140 mW) for 100 frames, 64 pixel X 64 pixel, 0.109 µm per pixel, scan speed 100 Hz, pixel dwell time 102.375 µs, line accumulation of 2.

Three sets of experiments were repeated at different imaging positions. Data analysis was done as described in Methods.

Supplementary Figure 2: Photo-bleaching properties of DBOV by excitation laser

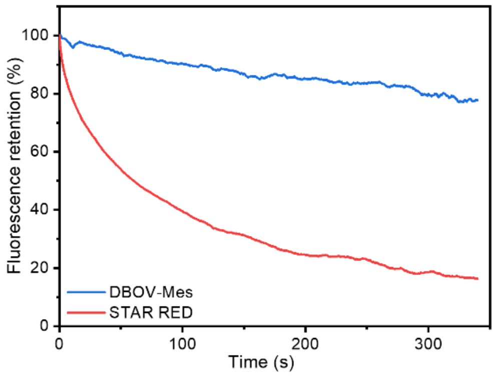

Photo-bleaching properties of DBOV-Mes and STAR RED as a function of the imaging time. Samples were dyes dropped on coverslips and measured in air after the solution evaporated. The measurements were performed with a Leica Stellaris 8 STED microscope. Series of Confocal images were acquired for comparison of photo-bleaching properties. Confocal images (500 frames) were acquired using an excitation wavelength of 610 nm at 20% laser power. Emission was collected across a range from 620 nm to 750 nm. Setting: 64 pixel X 64 pixel, 0.159 μm per pixel, pixel dwell time 102.375 μs, total collection time is 340.059 s.

Supplementary Figure 3: Representative line profiles from images in Fig. 1b-d

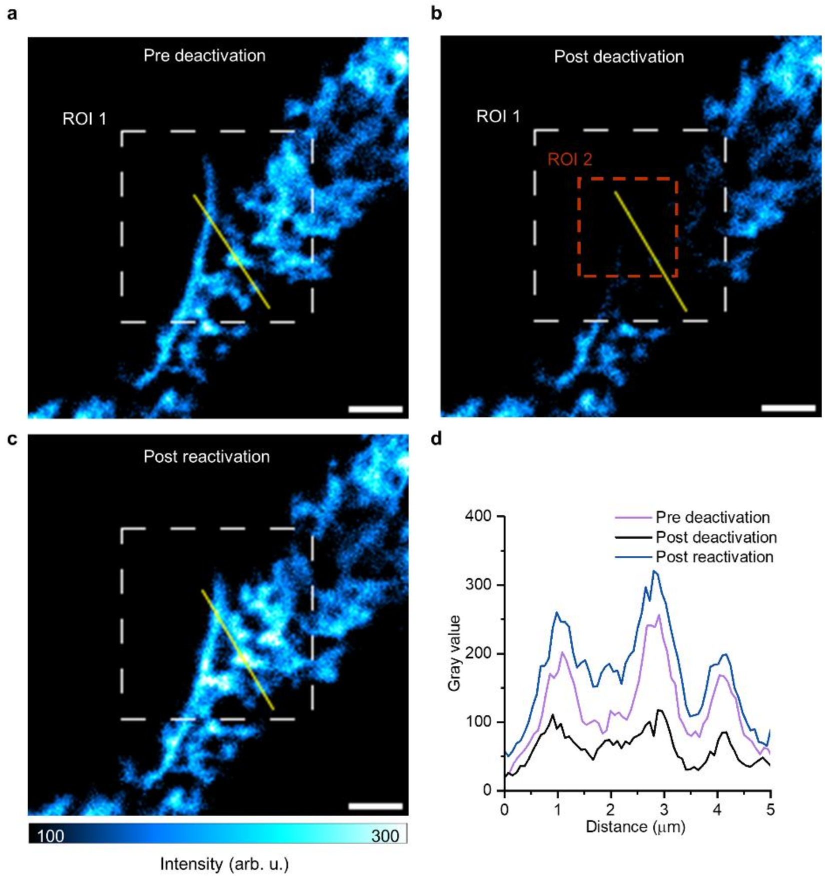

Representative line profiles from images in Fig. 1b-d. a-c, Confocal images shown before deactivation, after deactivation, and after reactivation as depicted in Fig. 1b-d. **d**, Representative line profiles corresponding to the yellow lines marked in the images. Scales, 2 µm. a-c share the same color bar which represents a linear scale.

Supplementary Figure 4: Possible ionization mechanisms

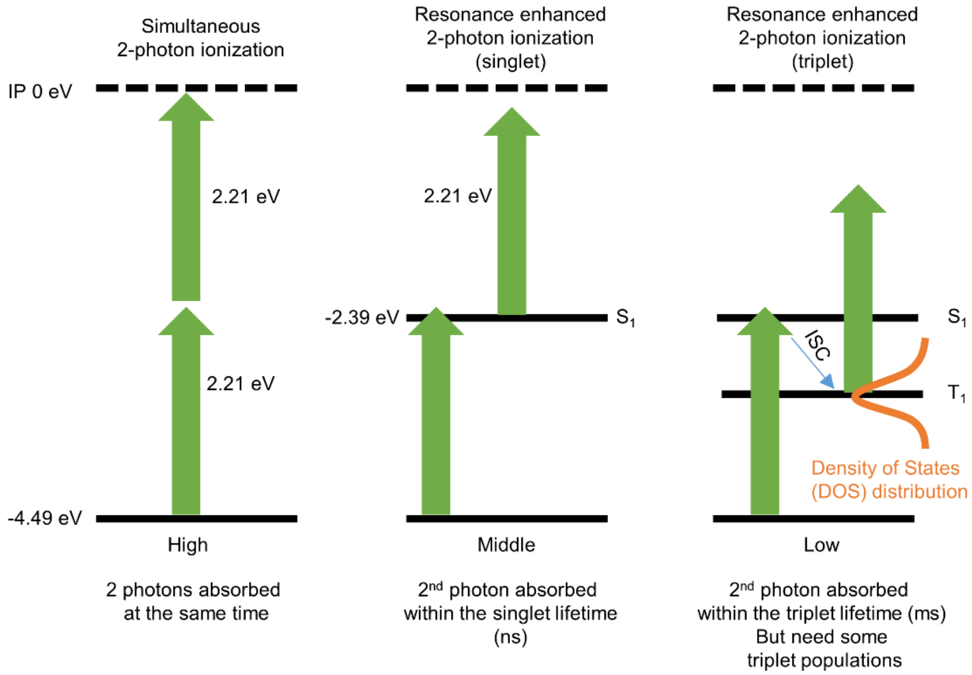

Schematic diagram shows possible ionization mechanisms. Resonance enhanced multi-photon ionization (REMPI) via S_1 or T_1 is observed in addition to simultaneous multi-photon ionization in organic materials. In REMPI, S_1 or T_1 is formed by the first photoexcitation, and before this excited state is deactivated, the next photon is absorbed to obtain energy above the ionization potential. In general, the lifetime of S_1 is nanoseconds and T_1 is microseconds to milliseconds, so the required excitation energy is lower than in simultaneous multi-photon ionization. It is difficult to distinguish between these processes, but considering the HOMO of DBOV-Mes and the laser wavelength, REMPI via S_1 would be the main possible mechanism.

Supplementary Figure 5: UV-vis-NIR absorption spectra of DBOV-Mes and DBOV-Mes$^{\bullet+}$

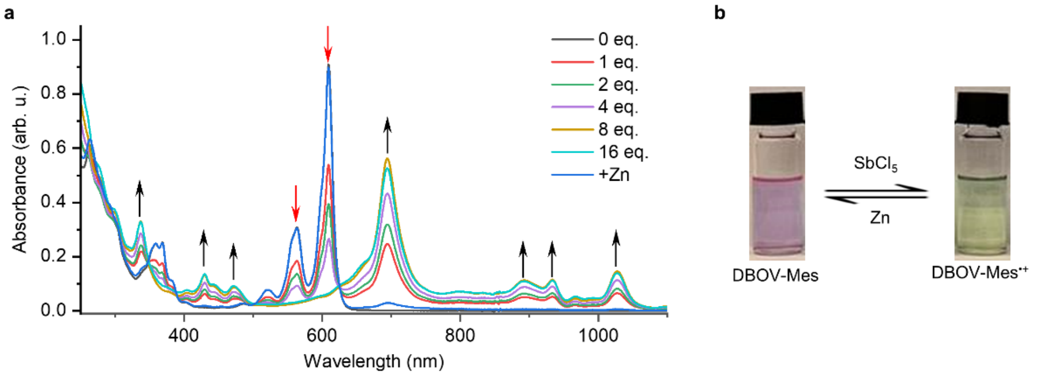

UV-vis-NIR absorption spectra of DBOV-Mes and DBOV-Mes$^{\bullet+}$. **a** UV-vis-NIR absorption spectra of DBOV-Mes and its radical cation by adding one electron oxidation reagent antimony chloride ($SbCl_5$) and neutral state DBOV-Mes be reduced with Zn powder. Red arrows indicate regions where the absorption decreases, while black arrows highlight areas where the absorption increases after the treatment. **b** color change of the dichloromethane solution of DBOV-Mes after oxidation with 16 equivalents of $SbCl_5$ and reduction with Zn powder. All spectra were measured in anhydrous dichloromethane. The oxidized species could be cleanly reduced back to the neutral state with Zn powder, indicating high stability of DBOV radical cation.

Supplementary Figure 6: EMAS measurement of a DBOV-Mes film

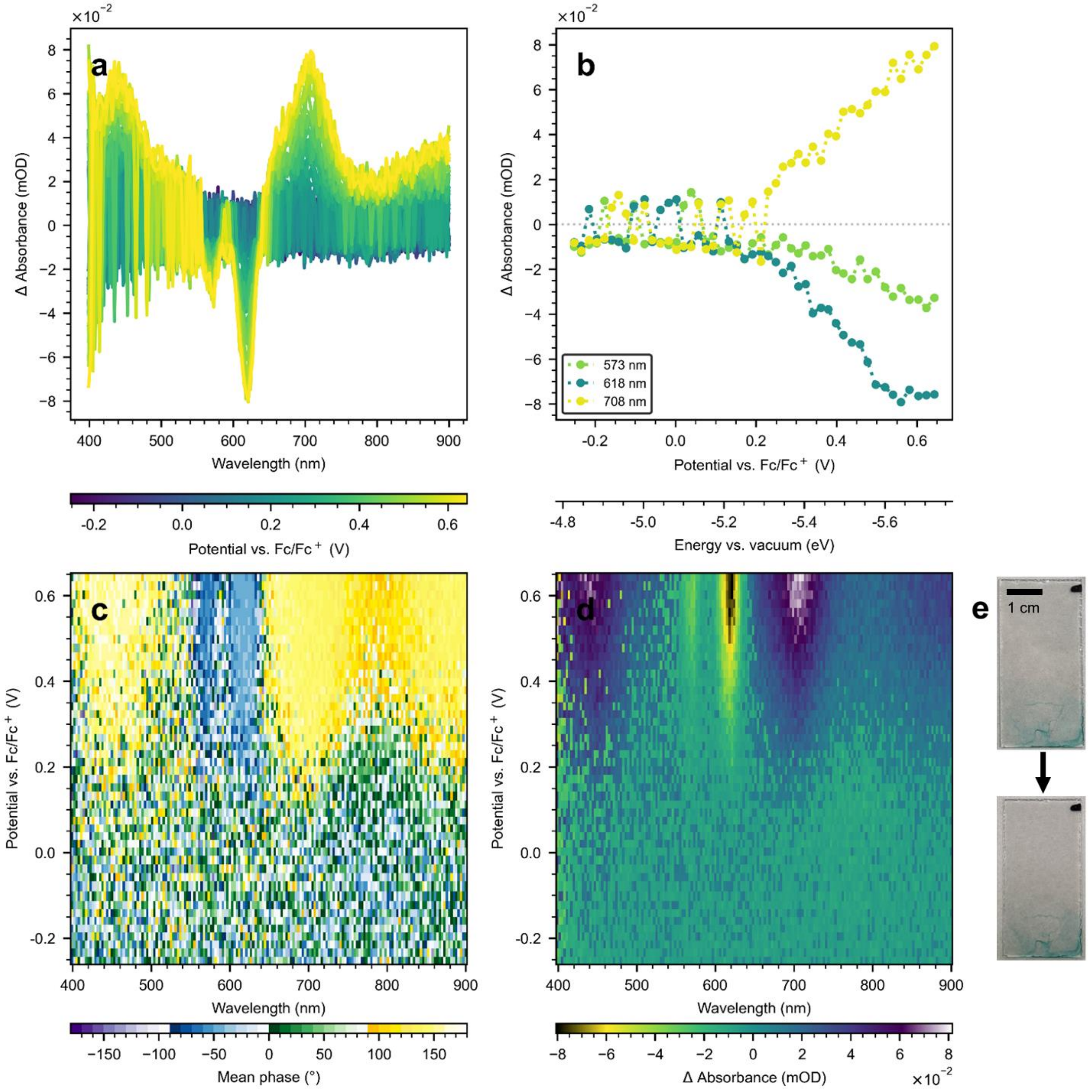

EMAS measurement of a DBOV-Mes film. a EMAS Spectrum of DBOV-Mes (50µL of 20 mM solution in toluene) dropcoated on FTO-coated glass showing the spectral changes of DBOV-Mes under electrochemical oxidation. The potentials for the ΔA-spectra are indicated by the color code. Upon oxidation new absorption peaks from 400 nm to 520 nm and from 660 nm to more than 900 nm are observed. These broad induced absorption bands are centered at 440 nm, 708 nm and above 800 nm. The positions of the observed bleaches of the characteristic absorption bands of DBOV-Mes

at 573 nm and 618 nm are in good agreement with the 0→0' and 0→1' transitions of DBOV-Mes as described by Coles, Lidzey et al.[1]. **b** Linecuts of the EMAS spectrum at specific, exemplary wavelengths give information about the onset potential of these absorption changes. Applying oxidative potentials above +(0.15 ± 0.1) V vs. Fc/Fc^+ causes the induced absorption at 708 nm and the bleaches at 573 nm and 618 nm, which we assign to the chemical transformation into the DBOV-$Mes^{\bullet+}$ radical. In **c** and **d** the whole EMAS dataset is shown as 2D contour plots of applied potentials vs. wavelengths. Here the phase relationships between the modulated potential and the modulated component in the optical signal (**c**) and the change in absorbance (**d**) is given by the respective color. Depicted in red are the wavelengths and potentials, at which induced absorption occurs. The bleaches are indicated in blue. The increasing absorptivity towards higher potentials shown for the oxidized state of DBOV-Mes reaches highest values at wavelengths of 440 nm and 708 nm. We believe a reactivation by lasers of similar wavelengths to be advantageous. Pictures of the DBOV-Mes coated FTO working electrode before and after the EMAS measurement show no visible damage to the sample (**e**) supporting the occurrence of a reversible change of the electrochemical structure of DBOV-Mes during the EMAS measurement.

Supplementary Figure 7: Calculated 2D STED intensity distributions

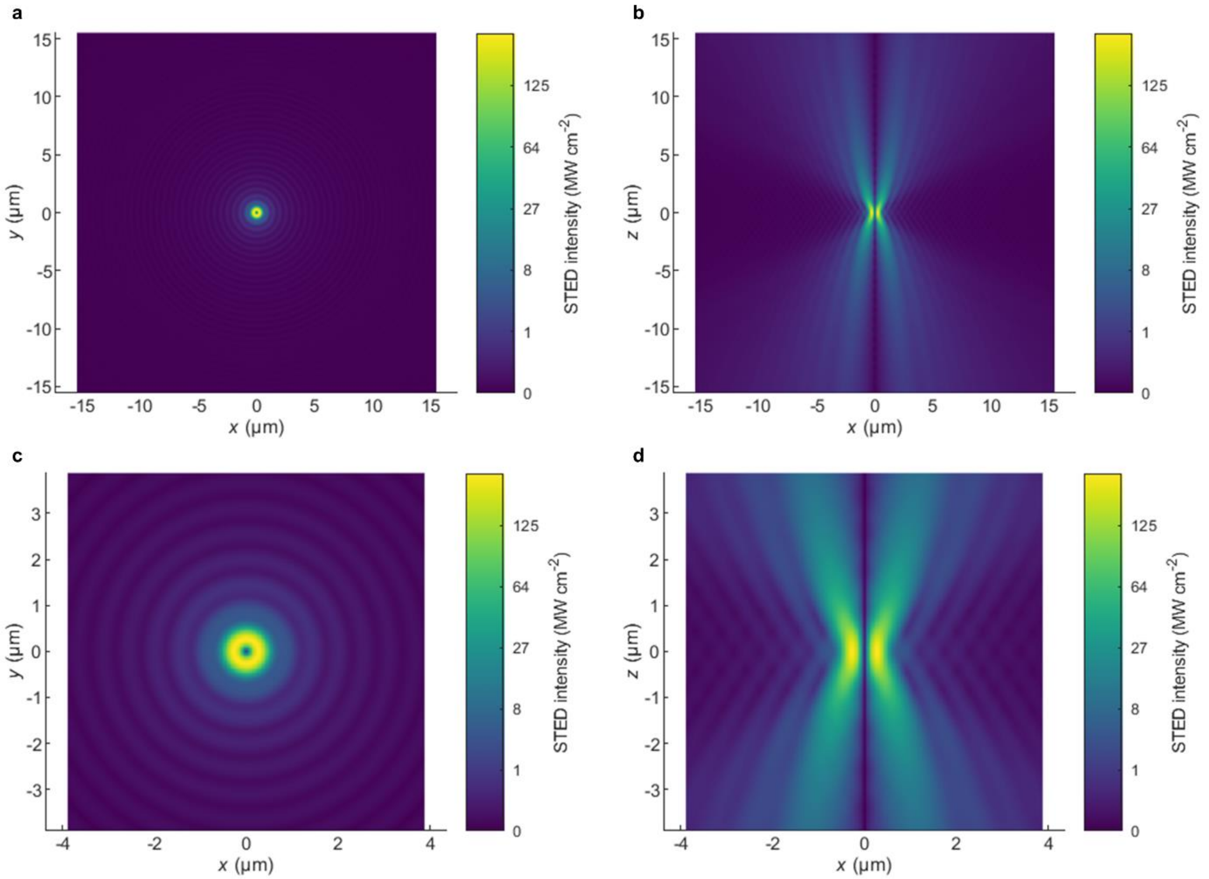

Calculated 2D STED intensity distributions. Calculated 2D STED intensity distributions in *xy* direction (**a**, **c**) and *xz* direction (**b**, **d**). **c** and **d** are the enlarged views of the central part of **a** and **b**. Color bars show the intensity in MW cm^{-2}, maximum value was set to 200 MW cm^{-2}. The color scale is non-linear, with a cubic transformation (x^3) applied to the colorbar tick labels.

Supplementary Figure 8: Measured leftover fluorescence ratio dependent on the confocal excitation laser density over time

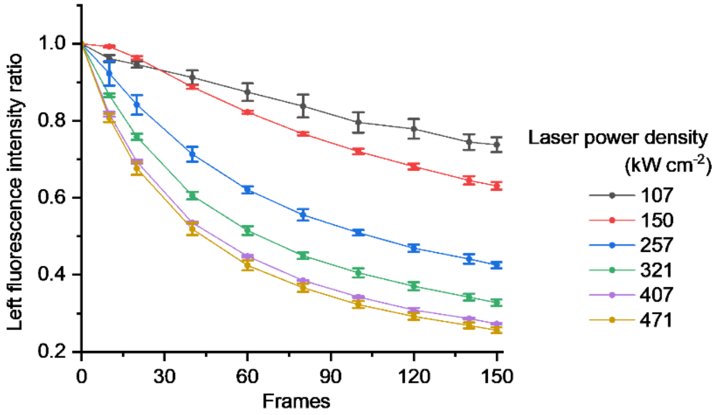

Measured leftover fluorescence ratio dependent on the confocal excitation laser density over time. Data of Fig. 2b obtained from Leica Stellaris 8 STED microscope. Experimental details see Supplementary Note 1.

Supplementary Figure 9: Plot (ln–ln) of fluorescence ratio against power density

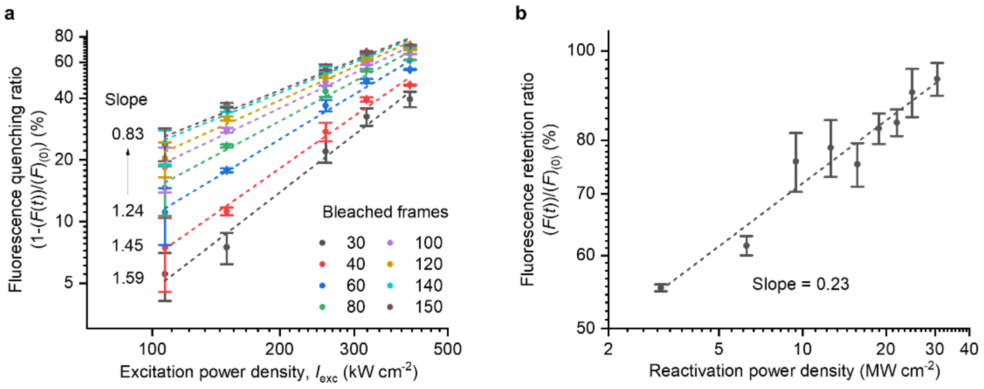

Plot (ln–ln) of fluorescence ratio against power density. a Plot (ln–ln) of quenching fluorescence ratio (1-($F(t)/F_0$)) against excitation power density (I_{exc}) over different imaging frames/time. The solid circles of different colors are the measured data, and the dash lines are the fitted linear curves with different slopes. **b** Plot (ln–ln) of leftover fluorescence ratio ($F(t)/F_0$) against STED power density (I_{STED}). $F(t)$: measured emission intensity at the frame time t; F_0: measured emission intensity at the frame time 0. Error bars in **a** and **b** represent standard deviations calculated from 3 or 4 measurements. Data analysis are described in Methods.

Supplementary Figure 10: Two-photon fluorescence under high STED beam peak intensity

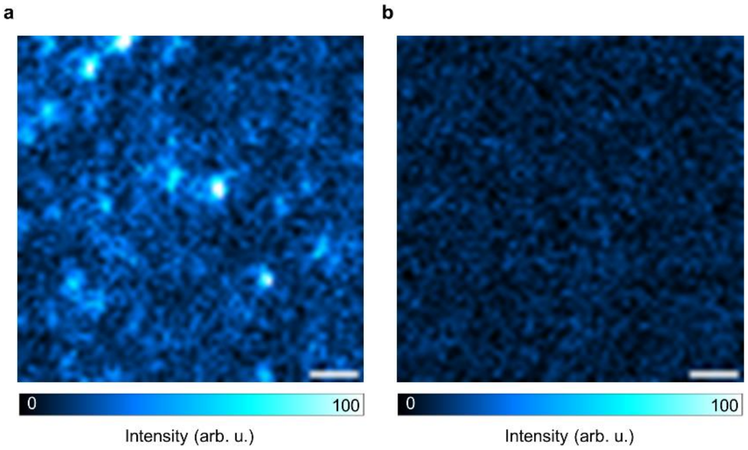

Two-photon fluorescence under high STED beam peak intensity. Images of DBOV-Mes on coverslip surface scanned by STED doughnut beam under 270 mW (**a**) and 140 mW (**b**). The color bars represent a linear scale. Setting: 64 pixel X 64 pixel, 0.109 μm per pixel, scan speed 100 Hz, pixel dwell time 102.375 μs, line accumulation of 2. Measured with Leica Stellaris 8 STED microscope.

Supplementary Figure 11: Fluorescence retention after different STED power densities scanning

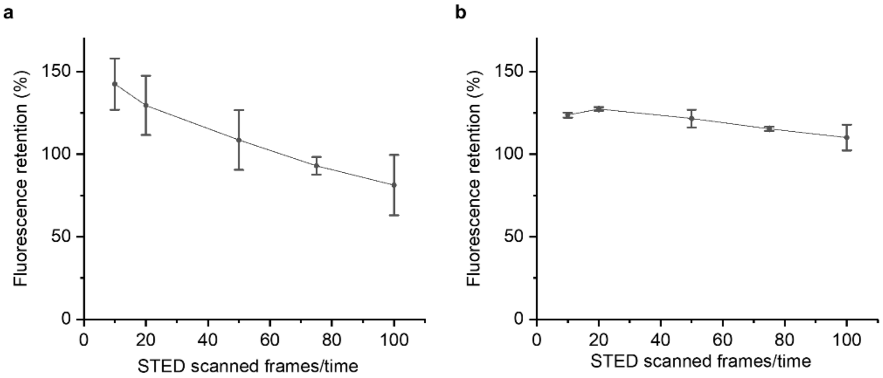

Fluorescence retention after different STED power densities scanning. Measured leftover fluorescence ratio over time (STED beam scanned frames). **a** 100% STED power (270 mW) was used. **b** 50% STED power (140 mW) was used. Experimental details are same as described in Supplementary Figure 1. Error bars in a, c and e represent standard deviations calculated from 3 or 4 measurements.

Supplementary Figure 12: Fluorescence retention after long term deactivation and reactivation cycles

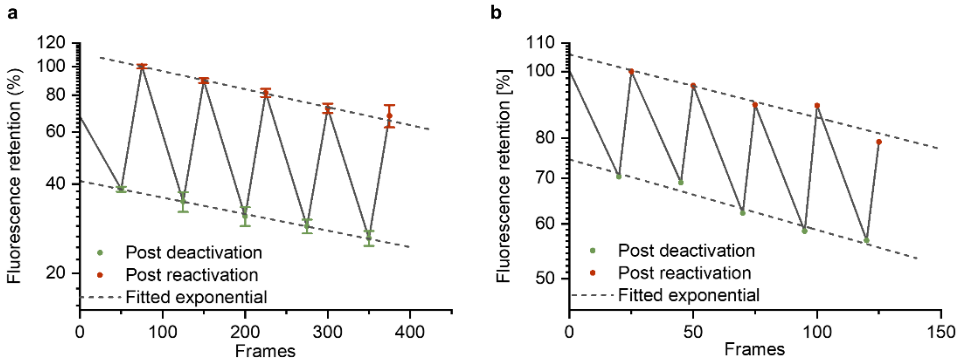

Fluorescence retention after long term deactivation and reactivation cycles. After repeated long term deactivation and reactivation, the fluorescence can still be recovered, but with an exponential decay.

For the cycles of deactivation-reactivation, images were acquired with the same settings as Fig. 2e except for the deactivation and reactivation frames in steps 2 and 4: **a** deactivation 50 frames, reactivation 25 frames; **b** deactivation 20 frames, reactivation 5 frames. Measured with Leica Stellaris 8 STED microscope. . Error bars in a, c and e represent standard deviations calculated from 3 or 4 measurements.

Supplementary Figure 13: Fluorescence decreases in long-term STED doughnut beam (only 775 nm) scanning

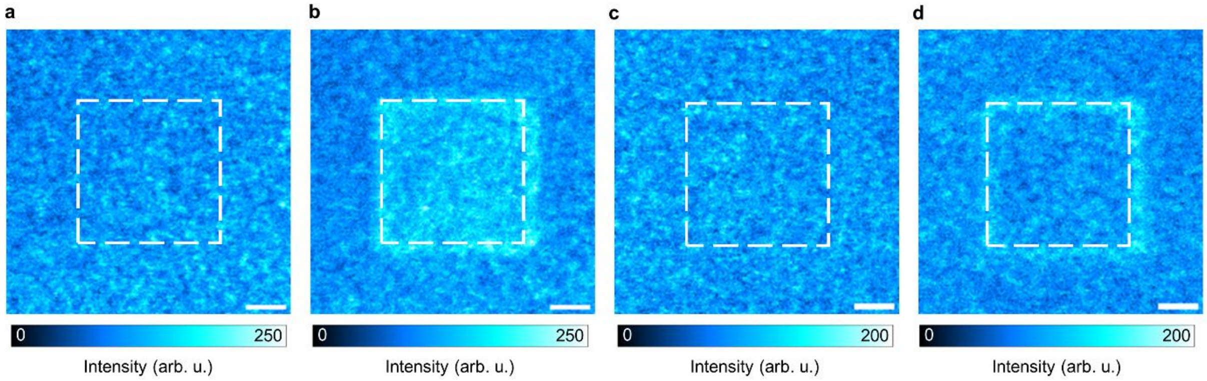

Representative confocal images of DBOV-Mes on coverslip surface before (**a**, **c**) and after (**b**, **d**) scanned by STED doughnut beam for 50 frames (**a, b**) and 100 frames (**c, d**). White dashed square region: STED beam scanned area. STED laser: 775 nm, 100% laser power of 270 mW. Scales, 2 μm. The color bar represents a linear scale. The fluorescence still kept 109% (average of 3 with standard deviations of 18%) after STED doughnut beam scanned for 50 frames. While after STED doughnut beam scanned for 100 frames, the fluorescence dropped to 81% (average of 3 with standard deviations of 18%), which might be induced by multi-photon effect. Measured with Leica Stellaris 8 STED microscope.

Note: The fluorescence intensity after scanned by the STED beam was even higher than in the first image because additional beam excitation is required to find the focal plane and select the target imaging area before imaging. Therefore, before taking the first confocal image, some DBOV-Mes molecules may already be in the fluorescent OFF state.

Images acquired by following the 3 steps (of one same imaging position):

Step 1 and 3, confocal images were acquired for measuring fluorescence intensity – before and after STED beam scanning. One confocal image using 561 nm (0.22 mW) as excitation, 512 pixel X 512 pixel, 0.076 μm per pixel, scan speed 100 Hz, pixel dwell time 15.3875 μs, line accumulation of 2, HyD X4 (600 nm – 748 nm).

Step 2, images (small, center of image of step 1) were scanned by only STED doughnut beam (775 nm, 270 mW) for 50 frames, 64 pixel X 64 pixel, 0.109 μm per pixel, scan speed 100 Hz, pixel dwell time 102.375 μs, line accumulation of 2.

Three sets of experiments were repeated at different imaging positions. Data analysis was done as described in Methods.

Supplementary Figure 14: STED imaging mode used to characterize the reactivation properties

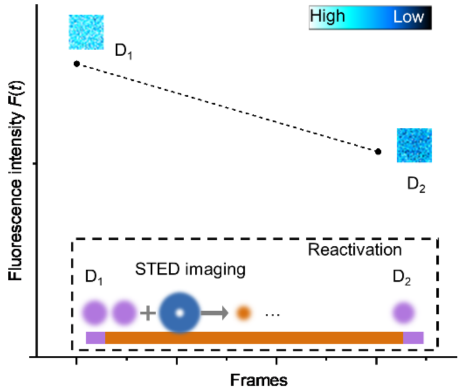

STED imaging mode used to characterize the reactivation properties. For studying the deactivation/reactivation effects of STED imaging, two confocal images were recorded by excitation, before (D_1) and after (D_2) STED imaging. The color bar represents a linear scale.

Supplementary Figure 15: Reactivation measured in air, H_2O and PBS

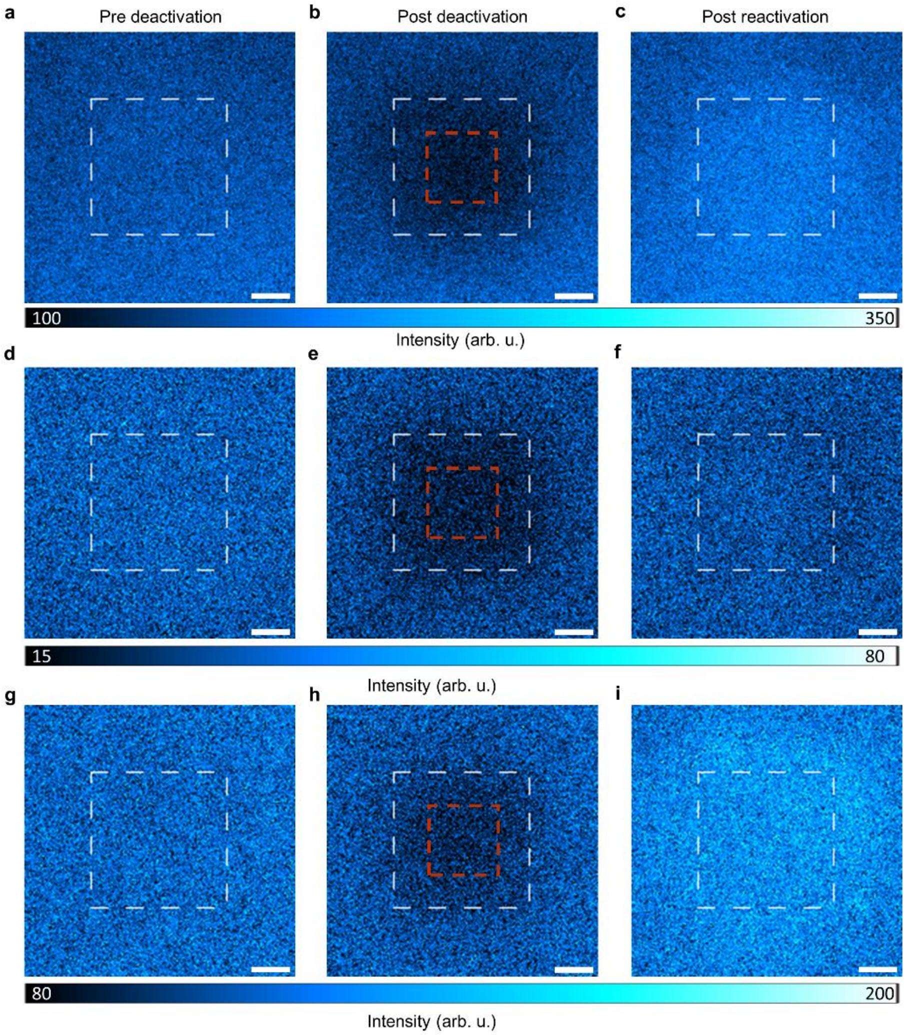

Images showing fluorescence reactivation measured in air, H_2O and PBS. Confocal images of DBOV-Mes on coverslip surface measured in air (**a**-**c**) H_2O (**d**-**f**) and PBS (**g**-**i**) before (**a**, **d**, **g**) and after (**b**, **e**, **h**) deactivation and after reactivation by STED beam (**c**, **f**, **i**). White dashed square region: deactivated area. Red dashed square region: STED beam scanned area. All the images have the same size. Scale bars, 2 μm. The color bars represent a linear scale. Measured with Leica Stellaris 8 STED microscope.

Supplementary Figure 16: Combined illumination of the excitation and STED beams has negligible photo-bleaching effect on the sample

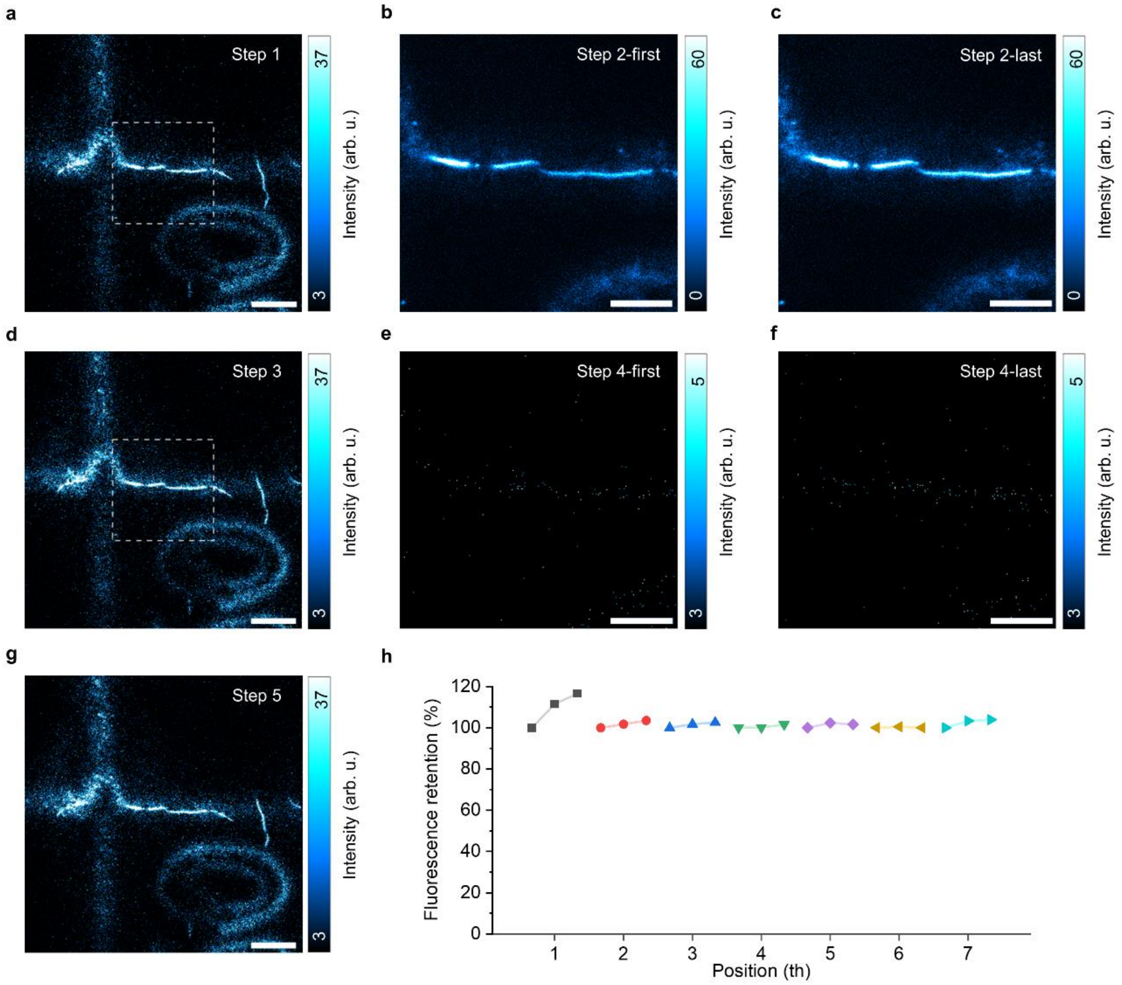

Combined illumination of the excitation and STED beams has negligible photo-bleaching effect on the sample. a-g Confocal images of the same area acquired by following the 5 steps: step 1, 3 and 5, one confocal image using 561 nm as excitation; step 2, co-excitation of area in dashed box by 561 nm and 775 nm (5 frames); step 4, scanning of area in dashed box by 775 nm laser (5 frames). **h** Fluorescence retention of the ROIs (same size as dashed box) in 7 different imaging positions. Each set has 3 points: first, step 1 (before co-excitation); second, step 3 (after co-excitation); third, (after 775 nm beam scanning). Scale bars, a, d, g: 10 μm; b, c, e, f: 5 μm. The color bars represent a linear scale. Images acquired from Zeiss Axio Examiner.Z1 microscope (with LSM 980 and Airyscan 2). Power of 561 nm laser measured under objective is 4 μW.

Supplementary Figure 17: Reactivation by NIR light at 730 nm

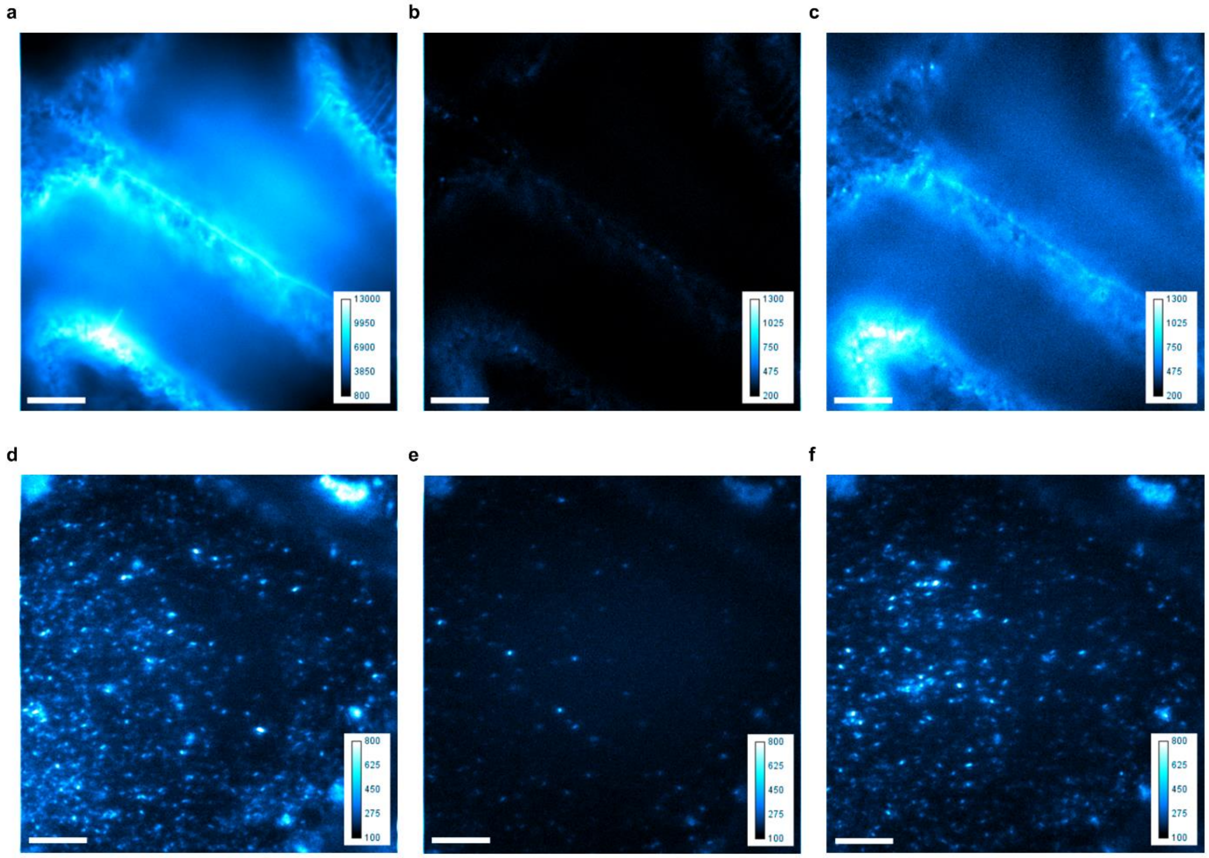

Reactivation by NIR light at 730 nm. Images acquired from Home-build widefield microscope. Widefield images of DBOV-Mes before (**a**, **d**) and after (**b**, **e**) deactivation and after reactivation by 730 nm light (**c**, **f**). **a**-**c**: DBOV-Mes in cracks of coverslip (μM); **d**-**f**: DBOV-Mes on coverslip surface (nM). Scale bars, 5 μm. This experiment was done with a home-built wide field microscope (see supplementary methods). The irradiance of about 4 kW cm^{-2} of 561 nm was used. Light from a 730 nm laser (30 mW, beam diameter 0.8 mm, ~ 6 W cm^{-2}) was used for reactivation. There are 3 steps in the reactivation experiments. Step 1, 10000 frames of widefield images with 561 nm for excitation were acquired with a 30 ms of camera exposure time. Step 2, turned on 730 nm light for 1 min. Step 3: Acquire another wide field image with 561 nm for excitation. Around 10% of the fluorescence is recovered by continuous low-power 730 nm laser.

Supplementary Figure 18: Reactivation by shorter visible light at 405 nm and spontaneously decay

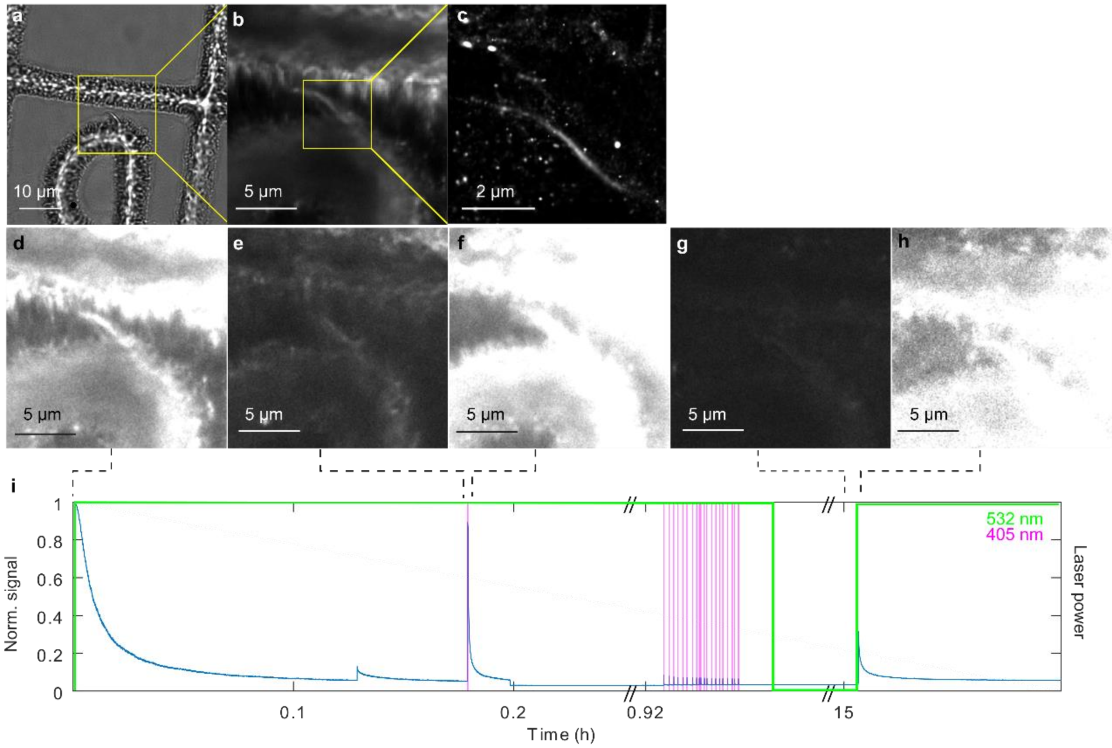

Reactivation by shorter visible light at 405 nm and spontaneously decay. Images acquired from Leica SR GSD microscope. Widefield images and time trace of fluorescence signal recovery in air with and without 405 nm laser. **a**-**c** Images of the crack region: **a** Bright-field image and the yellow rectangle indicates the ROI of the crack for the widefield image shown in **b**; **b** The graphene DBOV-Mes TIRF fluorescence image of the crack region; **c** The super-resolution image of the ROI shown as a yellow rectangle in **b**. **d**-**h** Representative fluorescence images at five different times. **i** Time trace of the fluorescence signal. Inset: **d**-**f**, Images were acquired under continuous excitation with 532 nm and recovery by the laser with a wavelength of 405 nm; **g** the sample was kept in dark covering by the housing of the microscope system overnight; **h** After about 14 hours, 32% of the fluorescence signals could be recovered.

Supplementary Figure 19: Photostability from two 3D STED imaging

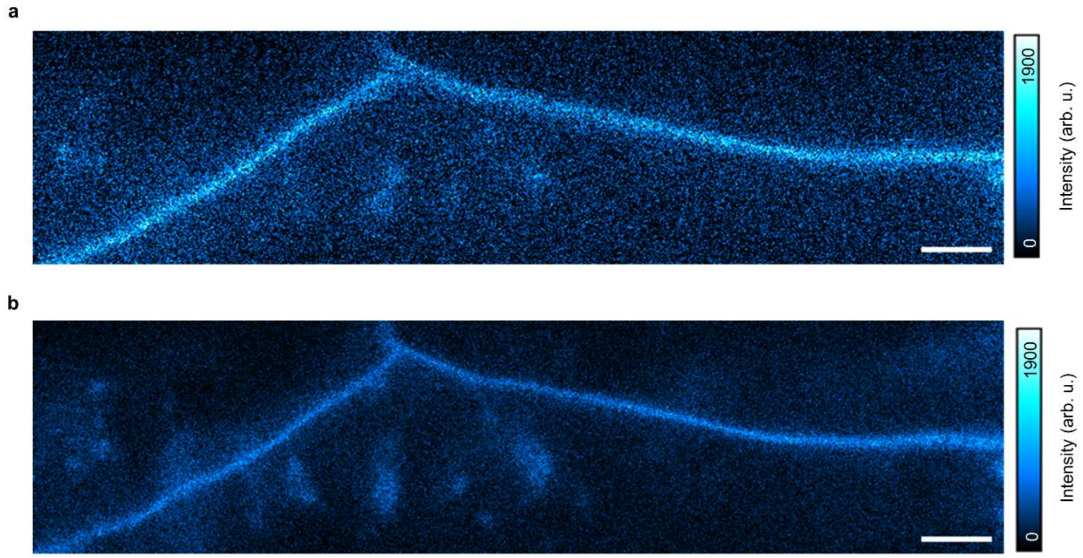

Photostability from two 3D STED imaging. Two measurements of 3D imaging over the similar imaging volume were performed. The data of the second measurement was plotted in Fig. 4f-h. Here we show the representative 2D STED images from two measurements. Upper image is from the first measurement of gridded structures in a glass substrate with DBOV-Mes (same imaging position as shown Fig. 4h). The image (**b**) is same as Fig. 4h but with adjusted contrast as shown in color bar. Scale bar: 1 µm. The color bars represent a linear scale. Settings for first measurement: 561 nm (AOTF setting: 40.52%) as excitation, 775 nm (AOTF setting: 86.03%) STED beam, 1024 pixel X 1024 pixel, 0.019 µm per pixel, 38 steps for z stack with 0.156 µm per step, scan speed 400 Hz, pixel dwell time 0.6 µs, line accumulation of 1, HyD 2 (600 nm – 748 nm). We note here that the main difference of settings to measure these two images is the number of averaged line scans – 1 for the upper image and 8 for the lower image. This should be the reason that these two images show different qualities.

To find the proper settings for STED imaging, we first tested with different settings on the same imaging area, and the images in Fig. 4f,g were obtained using the final selected parameters. The total imaging time for the entire 3D volume of Fig. 4f,g was over 73 min. Here, we compared the former measurement which has the similar settings with images in Fig. 4f,g. The representative 2D images at the same position of two measurements were selected out. The mean intensity of two 2D STED images was recorded to determine the leftover fluorescence ratio. The fluorescence retention was calculated by dividing the mean gray value of the same regions from two measurements. Seven regions of interest (ROIs) were selected as marked in Fig. 4h. Measured with Leica TCS SP8 STED microscope.

Supplementary Figure 20: Schematic diagram of liposome preparation

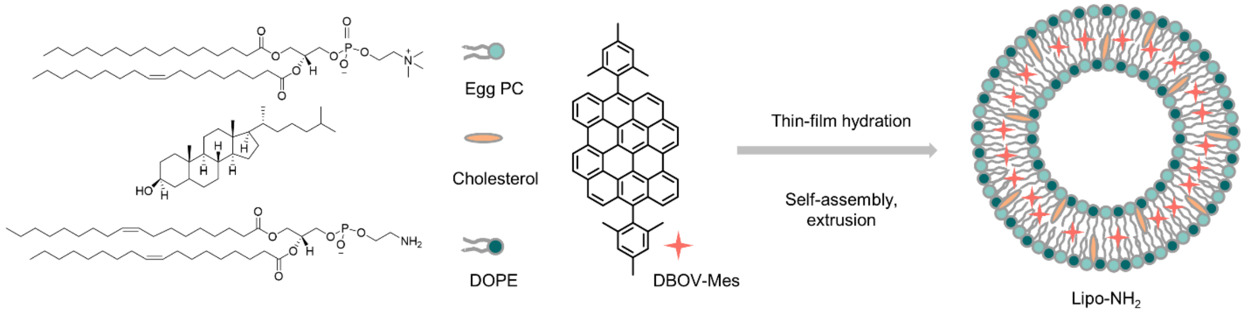

Schematic diagram of liposome preparation. The detailed protocol is described in Methods in main text.

Supplementary Figure 21: Size and zeta potential characterization of DBOV-Mes-labeled freshly prepared liposomes

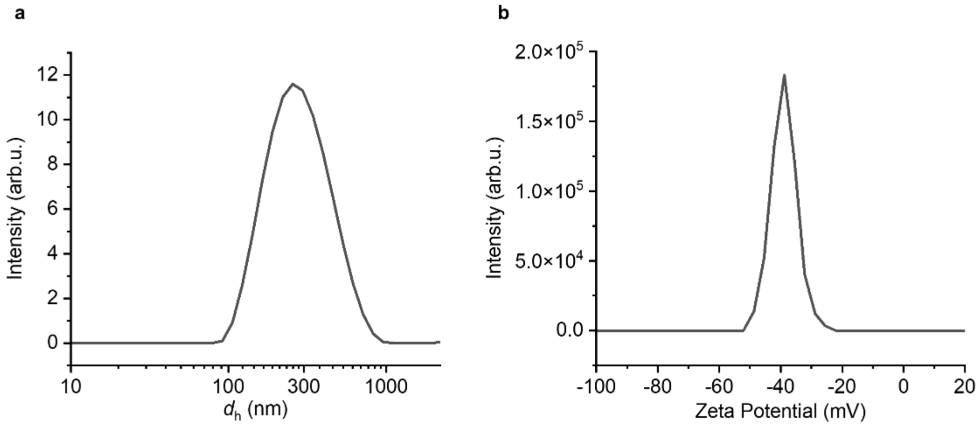

Size and zeta potential characterization of DBOV-Mes-labeled freshly prepared liposomes. a Dynamic light scattering result shows a mean size of 279 nm of the DBOV-Mes-labeled freshly prepared liposomes. **b** Average zeta potential is -40 mV ± 5mV.

Supplementary Figure 22: Schematic diagram of liposome immobilization

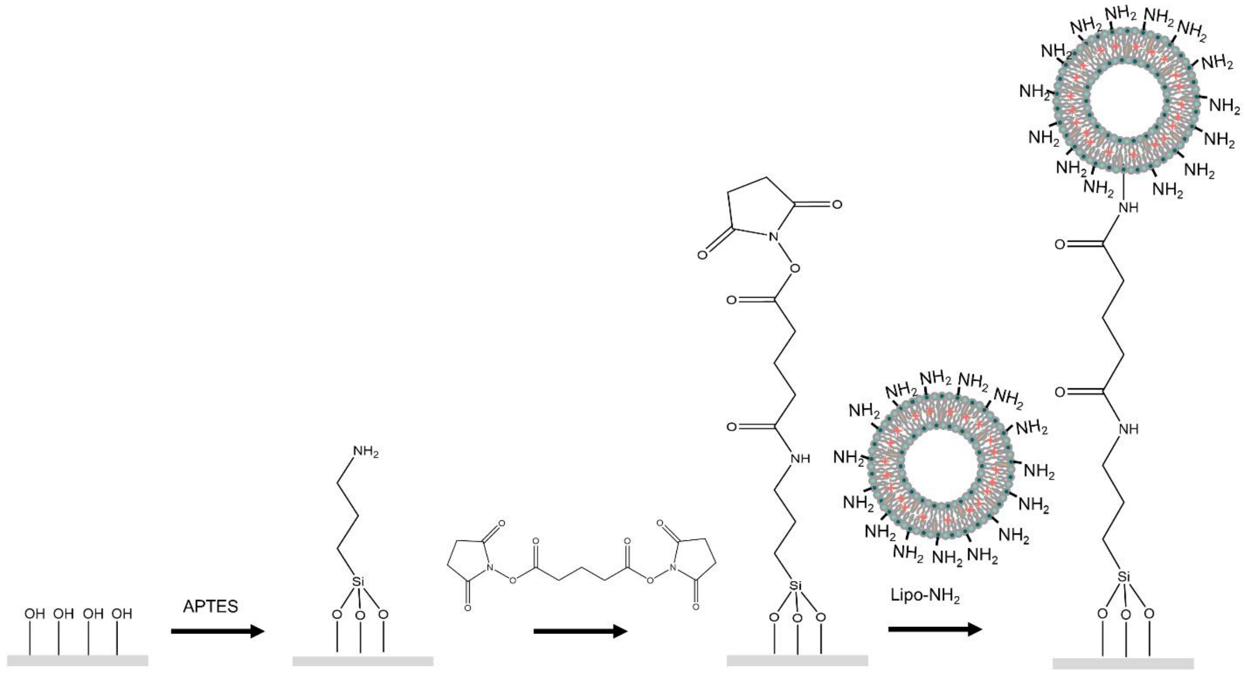

Schematic diagram of liposome immobilization on functionalized coverslip surface. The detailed protocol is described in Methods in main text.

Supplementary Figure 23: STED images of deformed liposomes

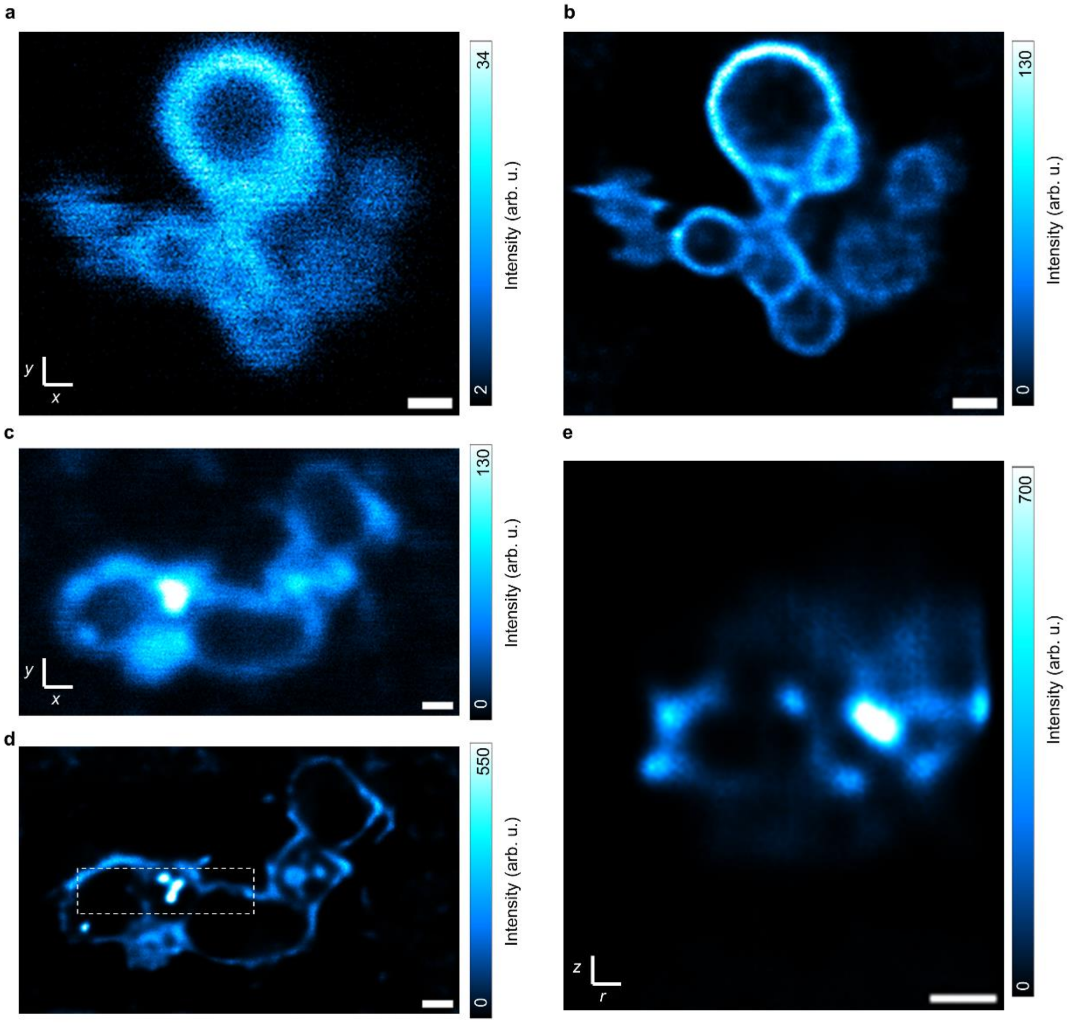

Confocal and 2D/3D-STED mode images of DBOV-Mes labelled LUVs and GUVs stored for more than one year. **a**,**b** 2D confocal (**a**) and STED (**b**) images of LUVs and GUVs. **c**-**e** 2D confocal (**c**) and STED (**d**) images of another imaging area of LUVs and GUVs and corresponding 3D STED (**e**) image of ROI in dashed box. Scale bars: 500 nm. All the color bar represents a linear scale. Measured with Abberior Expert Line microscope.

Supplementary Figure 24: Non-deconvolved STED images corresponding to Fig. 5

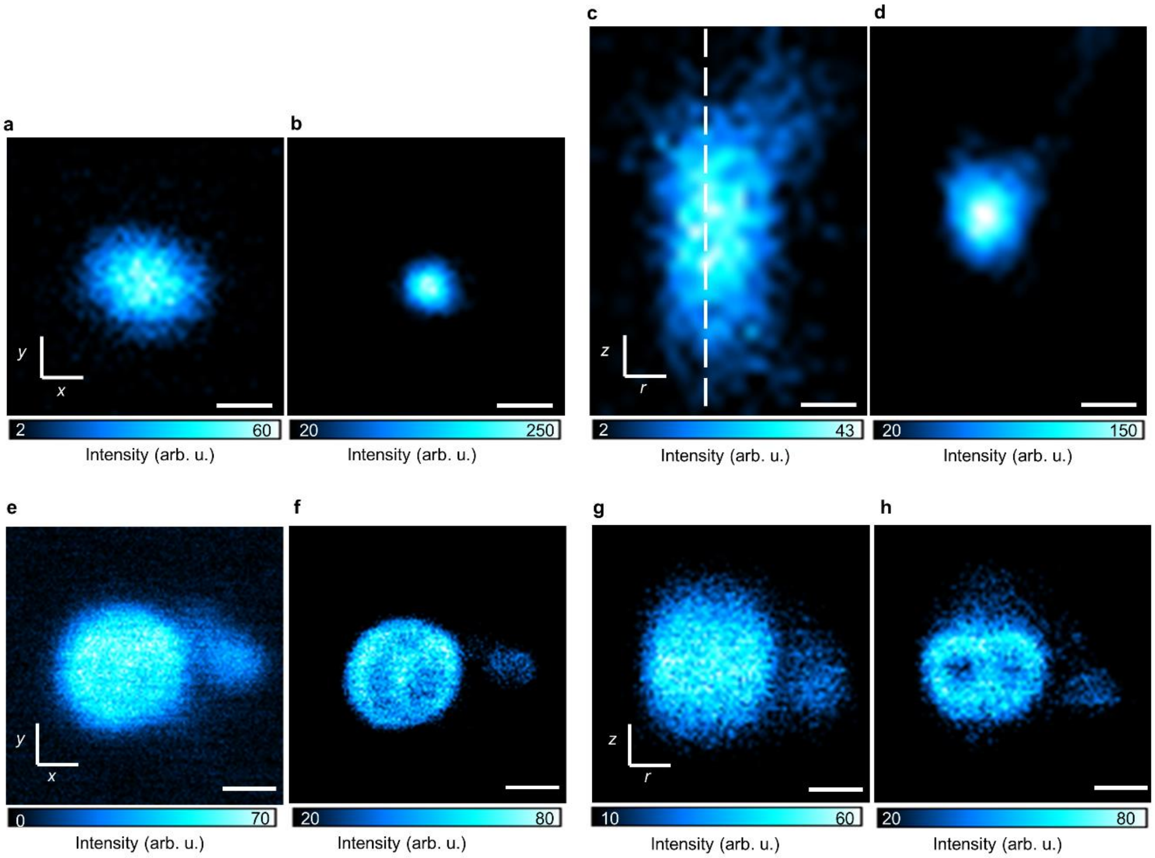

Non-deconvolved STED images corresponding to Fig. 5. 2D and 3D-3D (3D-STED beam depletion in both the xy plane and along the z-axis) Confocal and STED images of freshly prepared DBOV-Mes-labeled liposome. Scale bars, 200 nm (a-d) 500 nm (e-h). All the color bar represents a linear scale.

Supplementary references

1. Coles, D. M. *et al.* Strong Exciton–Photon Coupling in a Nanographene Filled Microcavity. *Nano Lett.* **17**, 5521–5525 (2017).
