# Supplementary material for: Reactivatable stimulated emission depletion microscopy using fluorescence-recoverable nanographene
Source: Nat Commun. 2025 Feb 4;16:1341. doi: 10.1038/s41467-025-56401-z (PMC11794581; doi:10.1038/s41467-025-56401-z)
Supplement: Supplementary file 2 — Description of Additional Supplementary Files [file 41467_2025_56401_MOESM2_ESM.pdf]

Description of Additional Supplementary Files

File Name: Supplementary Movie 1

Description: 3D confocal imaging of nanoscale cracks from the gridded structures in a glass substrate with DBOV-Mes

File Name: Supplementary Movie 2

Description: 3D STED imaging of nanoscale cracks from the gridded structures in a glass substrate with DBOV-Mes
